# The portrait of Dorian Gray: spontaneous expression of happiness is an invariant kinematic marker

**DOI:** 10.3389/fpsyg.2025.1546418

**Published:** 2025-07-24

**Authors:** Elisa Straulino, Cristina Scarpazza, Alessio Miolla, Andrea Spoto, Sonia Betti, Luisa Sartori

**Affiliations:** ^1^Department of General Psychology, University of Padua, Padua, Italy; ^2^Padova Neuroscience Center, University of Padua, Padua, Italy; ^3^Translational Neuroimaging and Cognitive Lab, IRCCS S. Camillo Hospital, Venice, Italy

**Keywords:** emotion expressions, kinematics, happiness, emotional induction, motor contagion

## Abstract

Crucial changes in the dynamical development of a facial display can characterize and distinguish spontaneous and posed facial expressions, a topic that has been poorly investigated. To assess spontaneous expressions of happiness, we presented video clips extracted from comedies showing hilarious scenes (Emotional Induction, Experiment 1) or videoclips showing smiling faces (Motor Contagion, Experiment 2). To induce posed expressions, we adopted the classic image of posed happiness from Ekman’s dataset. The results showed high consistency for spontaneous expressions of happiness, characterized by reduced amplitude, speed and deceleration peaks of the smile and lower eyebrow distance compared to posed expressions, for both methods of emotion induction. Overall, we demonstrated that high-definition 3-D kinematics of dynamic facial movements together with FACS coding can provide relevant details to characterize the syntax of dynamic facial displays, showing that spontaneous expression of happiness is an individual fingerprint, unaltered by mood induction procedures. However, spontaneous smiling varied at the individual level, influenced by participants’ predisposition to cognitively empathize with the movie protagonist. These findings are significant for emotion research, which has largely overlooked the impact of mood induction methods and their relationship with interindividual variability.

## Introduction

The Picture of Dorian Gray is a philosophical fiction novel by Oscar Wilde. Dorian Gray expresses the wish that his portrait may grow old and he, instead, remain young, charming and handsome forever. His wish comes true, and the more he lives a life of pleasure, the more the smile on his portrait is ruined, while Dorian himself never changes. In the last chapter of the novel, he notices that “in the eyes there was a look of cunning, and in the mouth the curved wrinkle of the hypocrite.” This critical passage reminds us of people’s ability to voluntarily or involuntarily modulate their emotional expressions (Ekman and Friesen, 1975; Reed et al., 2003; Schmidtmann et al., 2020; Etcoff et al., 2021; Zloteanu et al., 2021) which, in turn, depends on the existence of two anatomically separate pathways for the production of facial expressions: the Voluntary Pathway (VP) and the Involuntary Pathway (IP). In fact, spontaneous and posed smiles have different neural pathways: the contraction of mimic muscles related to genuine emotion originates from subcortical brain areas that provide excitatory stimuli to the facial nerve nucleus in the brainstem via extrapyramidal motor tracts, which often involve the concomitant contraction of the ocular orbicular muscles. In contrast, posed smiles are controlled by impulses of the pyramidal tracts from the motor cortex (Frank et al., 1993; Schmidt et al., 2006; Sidequersky et al., 2014). Therefore, we have different pathways for posed (i.e., voluntarily controlled) and spontaneous (i.e., involuntarily produced) facial displays (Ross et al., 2016). Moreover, facial expressions are organized predominantly along the horizontal facial axis (i.e., upper-lower areas). The upper-face muscles (eyes areas) are mainly controlled by subcortical (e.g., basal ganglia) and extrapyramidal systems, whereas the lower face (mouth area) is under voluntary control of the motor system (Gazzaniga and Smylie, 1990; Hopf et al., 1992; Ross et al., 2007a; Krippl et al., 2015). Two consequences follow from this: (i) facial blends of expressions might occur across the horizontal axis (i.e., eyes vs. mouth areas; Ross et al., 2016); and (ii) muscles of the upper face are innervated bilaterally, whereas muscles of the lower face are cross-innervated prevalently from the contralateral side (Morecraft et al., 2004; Ross et al., 2016). Therefore, small changes in the dynamical development of a facial display may characterize and distinguish genuine from posed facial expressions, a topic still poorly investigated using sophisticated 3-D methods (but see Sowden et al., 2021). Recent research has suggested that spontaneous and intentionally posed displays can, to some degree, be distinguished. For instance, Namba et al. (2017) conducted a study where they captured genuine facial reactions to emotion-inducing films and deliberately posed expressions by instructing participants to intentionally convey four emotions: surprise, amusement, disgust, and sadness. Analysis of these expressions by using the FACS revealed observable differences in appearance between spontaneous and posed facial displays. These findings were further confirmed in a study where 2-dimensional video tracking showed morphological disparities for disgust and dynamic distinctions for amusement and surprise (Namba et al., 2021). However, studies exploring the 3-dimensional dynamics of facial displays to understand how they unfold over time and space remain limited. This is crucial, as facial expressions involve complex configurations with high degrees of freedom, allowing for dynamic rearrangements of facial features within milliseconds. Compared to the voluntary control of limbs (e.g., reaching and grasping) (Castiello, 2005), voluntary control of the face is still poorly understood.

This is particularly crucial for the expression of happiness, the easiest facial expression to pose (Ekman et al., 1988). People pretend to smile for conveying enjoyment and positive feelings or reflecting politeness and affiliation during daily social interaction (Ekman and Friesen, 1975; Calvo et al., 2013). It has been argued that only a spontaneous smile produces crow’s-feet wrinkles — the so-called Duchenne marker (Ekman et al., 1988, 1990; de Duchenne Boulogne, 1990; Frank et al., 1993). An increasing amount of evidence is however demonstrating that the Duchenne marker is not a reliable indicator. In fact, crow’s-feet wrinkles could also be produced voluntarily by contracting the zygomatic major muscle in the absence of spontaneous happiness (Schmidt et al., 2006, 2009; Krumhuber and Manstead, 2009; Gunnery et al., 2013; Girard et al., 2021). A more rigorous approach and more consistent proofs are therefore necessary to characterize and distinguish spontaneous from posed facial expressions of happiness (e.g., Horic-Asselin et al., 2020).

Past research systematically analyzed muscle activation through the Facial Action Coding System (FACS; Ekman et al., 2002; Ekman and Friesen, 1978). A FACS coder decomposes an observed expression into a fixed number of specific Action Units (AUs; i.e., contraction or relaxation of distinct facial muscles). This approach, however, is time-consuming (see Snoek et al., 2023 for automated alternatives on 2D images) and is affected by conflicting definitions of dynamic parameters during feature extraction, causing inconsistencies in the literature (Guo et al., 2018). Another major drawback of the present literature is that studies typically investigated participants’ expressions while they observed static images of posed expressions (Krumhuber et al., 2013) and the method adopted to elicit facial expression was heterogeneous (Siedlecka and Denson, 2019; for a review, see Salas et al., 2012). The most widely used procedures adopt techniques such as posing facial expression (Laird et al., 1982; Ekman, 2007), or observing film clips (Gross and Levenson, 1995; Hagemann et al., 1999; Hewig et al., 2005; Philippot, 1993; Schaefer et al., 2010; for a review see Rottenberg et al., 2007). In the case of happiness, classical induction methods consisted in the observation of: (i) static images of posed happiness (Ekman and Friesen, 1976); (ii) scenes that made people smile (i.e., emotional induction, the transmission of emotions from one individual to another; Kavanagh and Winkielman, 2016; Prochazkova and Kret, 2017); or (iii) people dynamically expressing their emotion in a direct manner that promoted motor contagion (i.e., the automatic reproduction of the motor patterns of another individual; Hess and Fischer, 2014). To the best of our knowledge, no research compared the effectiveness of these different methods on the facial response from a kinematical perspective: Is one procedure more effective than another in eliciting a specific expression? So far, methods to induce facial expressions greatly differed across studies, and it is still unknown how these differences affected the final results (Krumhuber et al., 2021).

To recap, the lack of 3-D tools to investigate the dynamic characteristics of expression and the incongruencies in the elicitation methods are both sources of poor consensus on the temporal syntax and spatial morphometry of facial expressions of emotion (Frank et al., 2009). Here, we analyzed the unfolding of a facial expression with a high-definition 3-D optoelectronic system in conjunction with the concurrent validation of a professional FACS coder to overcome the cited methodological limitations and expand our understanding of how facial displays unfold over time and space.

The first aim of this study was to measure the performance of spontaneous and posed expressions of happiness to investigate possible differences across the horizontal axis (i.e., mouth vs. eyebrows). The second objective was to investigate different emotional induction methods to test their impact on the spontaneous expression of happiness. Finally, we correlated facial expressions with empathic traits to investigate the impact of each induction methods on participants with different components of empathy.

In Experiment 1 we presented two stimuli: (i) a videoclip extracted from a popular comedy–aimed at eliciting spontaneous expressions of happiness without showing smiling faces (i.e., indirect induction method; Miolla et al., 2022), and (ii) the classic picture of a smiling face from the Ekman and Friesen dataset (Ekman and Friesen, 1976) to induce participants to produce a voluntary expression of happiness. We expected differences in facial movements to emerge during posed expressions of happiness specifically in the lower part of the face, as it is innervated by the voluntary pathway. Experiment 2 was designed to test the effect of motor contagion on the kinematics of a spontaneous expression of happiness. For the spontaneous condition we adopted real-life YouTube videos in which ordinary people shot frontally manifested the expression of happiness, and the posed condition was the same as for the previous experiment. In general, we expected to replicate and confirm in both experiments the differences between spontaneous and posed expressions, due to the anatomical dual innervation. However, we expected differences to emerge for the spontaneous responses due to induction methods and interpersonal differences. In particular, we expected a correlation between the kinematic variables and the cognitive component of empathy in Experiment 1 (indirect method of happiness induction), and between the kinematic variables and the emotional component of empathy in Experiment 2 (direct method of happiness induction). To this end, we specifically chose the Interpersonal Reactivity Index (IRI; Davis, 1983) because it allows a multi-dimensional assessment of different empathy components (i.e., emotional and cognitive).

## General methods

The data for Experiments 1 and 2 were collected at the Department of General Psychology - University of Padua.

### Ethics statement

All Experiments were conducted in accordance with the Declaration of Helsinki and approved by the Ethics Committee of the University of Padova (protocol n. 3,580, 4,539). All participants were naïve to the purposes of the experiment and gave their written informed consent for their participation.

### Participants

We recruited 45 participants with normal or corrected-to-normal vision, naïve to the experimental design and study purpose. All participants were divided into two independent groups, one for each Experiment (1, 2 see below), so that the two experiment did not suffer from non-independency.

### Apparatus

**Figure 1:**
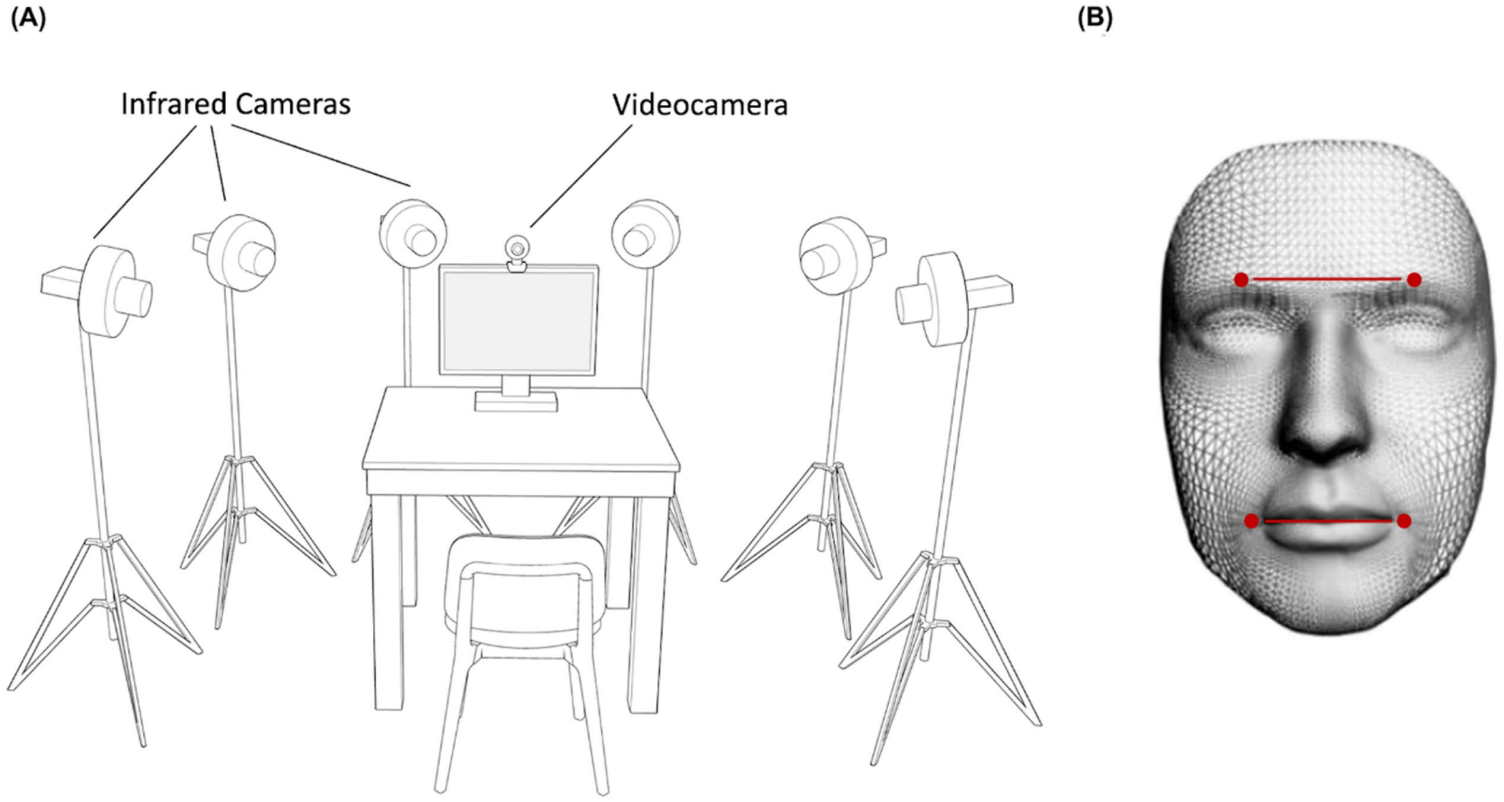
Experimental set up and Clepsydra model. Participants sat on a table in front of a computer screen with a video camera positioned above the monitor and six infrared cameras arranged in a semicircle around the table **(A)**. These cameras captured the relative position of four infrared reflective markers placed on the participant’s face and taped to the left and right Eyebrows, and to the left and right Cheilions **(B)**.

Participants were tested individually in a dimly lit room. Their faces were recorded frontally with a video camera (Logitech C920 HD Pro Webcam, Full HD 1080p/30fps) positioned above the monitor for the FACS validation procedure. Six infrared cameras (sampling rate 70 Hz), placed in a semicircle at a distance of 1–1.2 meters from the center of the room (Figure 1A) captured the relative position of four infrared reflective markers (3 mm diameter) applied to the face of participants. Capitalizing on the Clepsydra model recently developed by our laboratory (Straulino et al., 2023b), markers were taped to the left and right Eyebrows, and to the left and right Cheilions (Figure 1B). Facial movements were recorded using a 3-D motion analysis system (SMART-D, Bioengineering Technology and Systems [B|T|S]). The coordinates of the markers were reconstructed with an accuracy of 0.2 mm over the field of view. The standard deviation of the reconstruction error was 0.2 mm for the vertical (Y) axis and 0.3 mm for the two horizontal (X and Z) axis. The stimuli presentation was implemented on a monitor using E-prime V2.0. The dimension of each stimulus was 1,024 × 768 pixels displayed on a 22-inch monitor (resolution: 1280 × 1,024 pixels, refresh rate 60 Hz, color depth: 32 bits).

### Procedure

**Figure 2:**
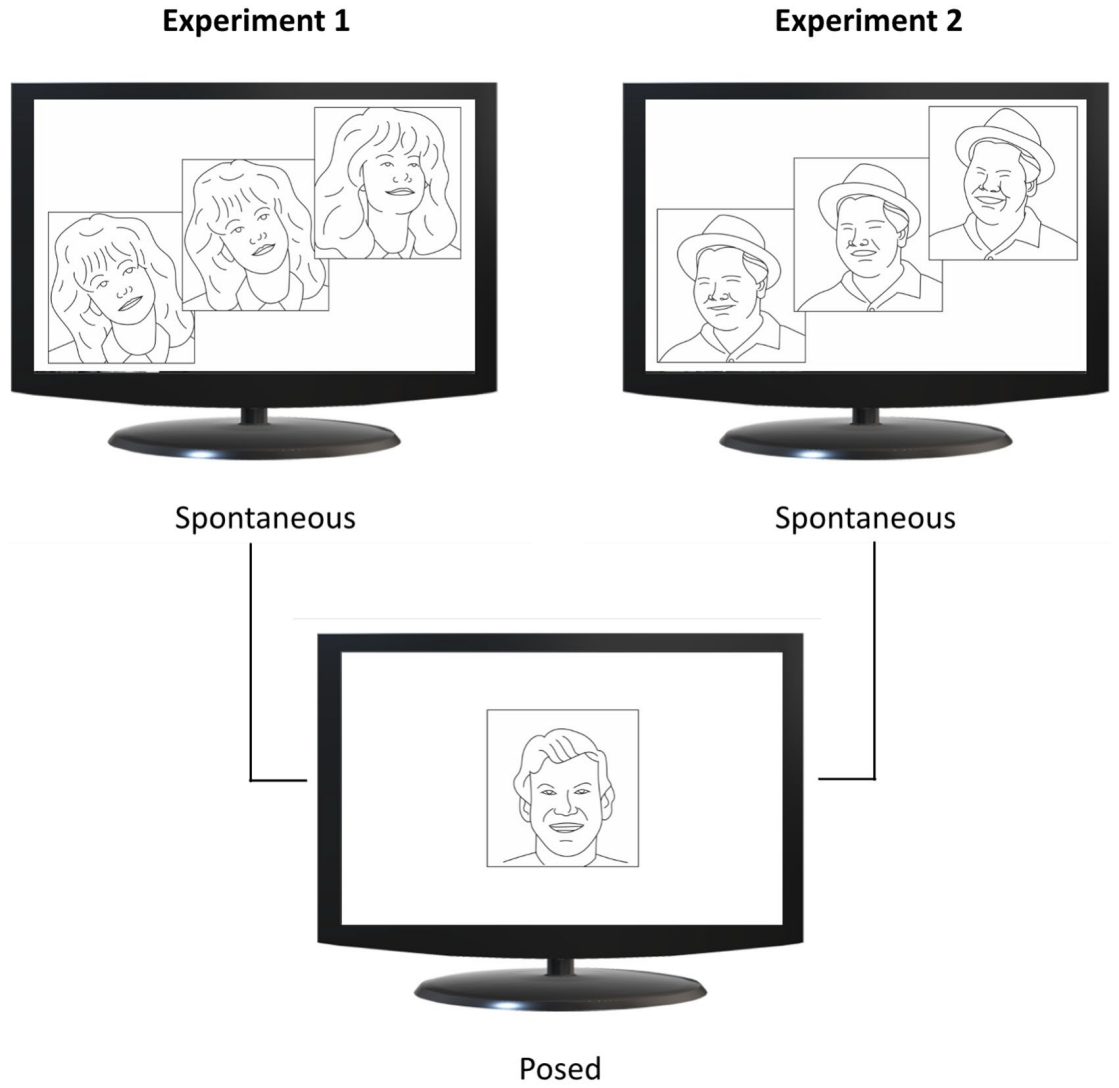
Experimental design. Spontaneous conditions are shown in the upper half of the figure, and posed conditions in the lower half. In Experiment 1 (upper left panel), participants viewed video clips from comedy scenes (upper image) and a static image of happiness (lower image). In Experiment 2 (upper right panel), participants viewed videos showing happy faces (upper image) and the same static image of happiness adopted in Experiment 1 (lower image). Image adapted from Straulino et al. (2023b), CC BY 4.0.

Each participant underwent a single experimental session (Experiment 1 or 2) lasting approximately 20 min. They were seated in a height-adjustable chair in front of a monitor (40 cm from the edge of the table) and were free to move while observing the stimuli displayed on the monitor (Figure 1A). Facial movements were recorded during two conditions: (i) Spontaneous condition, in which participants watched happiness-inducing videos and reacted freely; (ii) Posed condition, in which participants produced a voluntary expression of happiness three times, while a posed image of happiness was shown on the monitor for 60 s (Figure 2). The posed condition was the same for both experiments to allow inter-individual comparison across them (see Comparison analysis paragraph). Participants were not given instructions on the duration of the expression, which they managed at their own will. This procedure was aimed at generating expressions without forcing the participants to respect time constraints (Miolla et al., 2022). All the stimuli adopted for the Spontaneous condition (Experiments 1 and 2) contained sounds of adult voices. A neutral blank screen was presented between one stimulus and another (Inter Stimulus Interval = 10 s). As concerns the order of recording condition, the spontaneous condition was followed by the posed condition for all participants to keep the purpose of the experiment hidden during the spontaneous block (Perusquía-Hernández et al., 2019; Sowden et al., 2021). In addition, in order to assess the emotional and cognitive components of empathy, each participant completed the Italian validated version of the Interpersonal Reactivity Index (IRI, Albiero et al., 2007; Davis, 1983; see below).

### Interpersonal reactivity index

The Italian version of the Interpersonal Reactivity Index (IRI; Albiero et al., 2007), is a 28-item self-report questionnaire to measure empathy answered on a 5-point Likert scale ranging from 1 = “does not describes me well” to 5 = “describes me very well.” The IRI has four different subscales, each made up of seven different items, covering both the cognitive and emotional components of empathy: (i) perspective taking (PT): the tendency to spontaneously adopt the psychological point of view of others; (ii) fantasy (FS): the tendency to transpose themselves imaginatively into the feelings and actions of fictitious characters in books, movies, and plays; (iii) empathic concern (EC): the feelings of sympathy and concern for unfortunate others; (iv) personal distress (PD): the feelings of personal anxiety and unease in tense interpersonal settings. The component of Cognitive empathy is assessed by means of the subscales PT and FS (i.e., the ability to adopt another person’s point of view and identify with fictional characters). The component of Emotional empathy is assessed by means of the subscales EC and PD (i.e., the ability to produce self-oriented and other-oriented emotional responses like sympathy).

### Expression extraction and FACS validation procedure

All repeated expressions of happiness produced during the observation of each video were included in the analysis. A two-step procedure was adopted to ensure a correct selection of each expression. A certified FACS coder (i.e., author A. M.) manually identified all the single epochs – the beginning and end of each smile –according to the FACS criteria (e.g., Action Units 6 and 12, the Cheek Raiser and the Lip Corner Puller). The kinematic analysis then automatically identified the beginning and end of the smiles within each epoch using the cross-reference algorithm on the threshold velocity of the Cheilion (Straulino et al., 2023b). The two results were compared and corrected for errors or missing data to obtain a 100% match. The manual FACS coding has a strong concurrent validity with the automated FACS coding, thus denoting the reliability of the method (De la Torre et al., 2011; Hamm et al., 2011). However, we chose to combine kinematic analysis with manual rather than automated FACS coding because, as reported in a recent paper (Le Mau et al., 2021), it continues to outperform automated coding.

### Data analysis

#### Kinematic 3-D tracking

Following kinematic data collection, each trial was individually checked for correct marker identification. The SMART-D Tracker software package (Bioengineering Technology and Systems, B|T|S) was employed to automatically reconstruct the 3-D marker positions as a function of time.

#### Kinematic feature extraction

To investigate spatial, velocity and temporal key kinematic parameters in both the upper and lower face, we considered the relative movement of two pairs of markers:

- Lower part of the face:
  - Left and Right Cheilions (CH)
- Upper part of the face:
  - Left and Right Eyebrows (EB)

**Figure 3:**
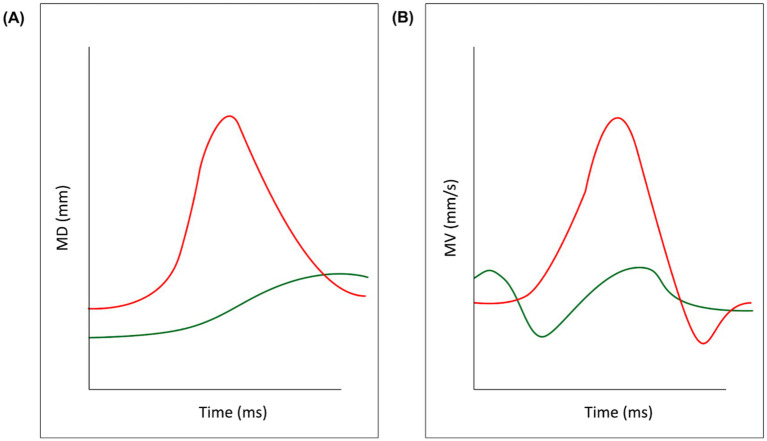
Key kinematic variables. Data from a representative participant in the experimental sample have been outlined for illustrative purposes. The red line refers to the two mouth markers (left and right Cheilion) while the green line refers to the two eyebrow markers. Panel **(A)** depicts the Maximum Distance between the two markers and panel **(B)** depicts their Maximum Velocity.

We selected the minimum number of markers adopted in the literature as a common denominator to compare our findings with previous results. In addition, the choice of the position of the four markers (mouth and eyebrows) was made to consider possible cultural biases in future studies, i.e., the tendency of East Asian people to place more emphasis on eye expression and that of Western people to focus on the mouth (Fox, 2020). Each expression was analyzed from the onset point to the apex (i.e., the peak). Movement onset was calculated as the first time point at which the mouth widening speed crossed a 0.2 mm/s threshold and remained above it for longer than 100 ms. Movement offset was considered when the lip corners reached the maximum distance (i.e., the time at which the mouth widening speed dropped below the threshold of 0.2 mm/s). Movement time was calculated as the time interval between the onset and the offset of the movement. The classical kinematic parameters for extracting spatial, velocity and temporal components of movement were then computed on each pair of markers of the mouth and eyebrows (Figure 1B):

- Spatial parameters:
  - Maximum Distance (MD; Figure 3A): the maximum distance reached by the 3-D coordinates (x,y,z) of two markers.
  - Delta Distance (DD): the difference between the maximum and the minimum distance reached by two markers, to account for functional and anatomical differences across participants.
- Velocity parameters:
  - Maximum Velocity (MV; Figure 3B): the maximum velocity reached by the 3-D coordinates (x,y,z) of each pair of markers.
  - Maximum Acceleration (MA, mm/s^2^): the maximum acceleration reached by the 3-D coordinates (x,y,z) of each pair of markers.
  - Maximum Deceleration (MDec, mm/s^2^): the maximum deceleration reached by the 3-D coordinates (x,y,z) of each pair of markers. Here it is reported in absolute value for graphical purposes.
- Temporal parameters:
  - Time to Maximum Distance (TMD%): the proportion of time at which a pair of markers reached a maximum distance, calculated from movement onset.
  - Time to Maximum Velocity (TMV%): the proportion of time at which a pair of markers reached a peak velocity, calculated from movement onset.
  - Time to Maximum Acceleration (TMA%): the proportion of time at which a pair of markers reached a peak acceleration, calculated from movement onset.
  - Time to Maximum Deceleration (TMDec%): the proportion of time at which a pair of markers reached a peak deceleration, calculated from movement onset.

### Statistical analysis

Behavioral data were analyzed using JASP version 0.16 (JASP Team, 2022) statistical software. Data analysis for each experiment was divided into three main parts: the first one was aimed at testing if facial motion differs across the horizontal axis (i.e., mouth vs. eyebrows) for spontaneous and posed emotional expressions; the second part was aimed at exploring the correlation between IRI and kinematics; the third part was aimed to explore differences in the induction method across the two experiments.

The first part of the analysis consisted in fitting Linear Mixed Effect Models on the lower and upper pairs of markers (i.e., CH, EB). As dependent variables (y), we considered the average value for each kinematic parameter described in the Data Analysis section; as independent variable (x), we considered the Condition (Spontaneous vs. Posed), which was computed as Fixed Effect. A random intercept was added to account for inter-individual differences. The Volk-Selke Maximum p-Ratio on the two-sided *p*-value was computed too, in order to quantify the maximum possible odds in favor of the alternative hypothesis over the null one (*VS-MPR*; Sellke et al., 2001). In addition, R-squared Conditional, that is the variance explained by the fixed and the random effects together over the total (expected) variance of the dependent variable, was included in the analysis. The analysis did not detect the presence of outliers. For the second part of statistical analysis, we performed a correlation analysis using the Pearson correlation coefficient (*p* < 0.05 Bonferroni corrected). Finally, to explore the possible differences triggered by different induction methods in the expression of happiness, we conducted a Linear Mixed Effect Model with Experiment (1, 2) and Condition (Posed, Spontaneous) as fixed effects and Individuals as random effects. For all statistical analyses, a significance threshold of *p* < 0.05 was set and Bonferroni correction was applied to *post-hoc* contrasts. Sample size was determined by means of GPOWER 3.1 (Erdfelder et al., 1996) based on previous literature. Since we used repeated-measures design, we considered an effect size *f* = 0.25, alpha = 0.05 and power = 0.8. The projected sample size needed with this effect size was *N* = 20 for within group comparisons in each experiment. This sample size allowed for post-hoc comparisons, assuming alpha 0.05 and with a power 1-beta of 0.8. Due to the exclusion of three participants, we subsequently conducted a *post hoc* power analysis and found that, even assuming small effect sizes, the achieved power remained high (i.e., >0.95). All data are available in the Supplementary materials section.

## Experiment 1

### Participants

Twenty participants (13 females, 4 males) aged between 21 and 32 years (*M*_age_ = 24.75, *SD* = 3.04) were recruited. Three participants were subsequently excluded due to 3-D tracking problems.

### Stimuli

For the Spontaneous condition, we selected *N* = 2 emotion-inducing videos from a recently-validated dataset structured to elicit genuine facial expressions (Miolla et al., 2022). Videoclips were extracted from popular comedy movies in which actors produced hilarity without showing smiling faces (e.g., jokes by professional comedians). Videoclips lasted an average of 2 min and 55 s (video 1 = 3 min 49 s; video 2 = 2 min 2 s), therefore not exceeding 5 min in accordance with the recommended maximum duration for emotional videos (Rottenberg et al., 2007). Each video was presented once without repetition to avoid possible habituation effects. Participants rated the intensity and the valence (computerized version of the Self-Assessment Manikin – SAM; Bradley and Lang, 1994) of the emotion felt while watching the video at the end of each presentation to assess the efficacy of the emotion induction method. Both stimuli were judged to have above-average intensity (7) and valence (7) on a 9-point Likert scale, where 1 indicated “not at all” and 9 indicated “very much.”

### Results

Participants performed a range of 3–5 expressions of happiness in the Spontaneous condition and three in the Posed condition.

#### Linear mixed effect models: spontaneous vs. posed lower part of the face–Cheilions

**Figure 4:**
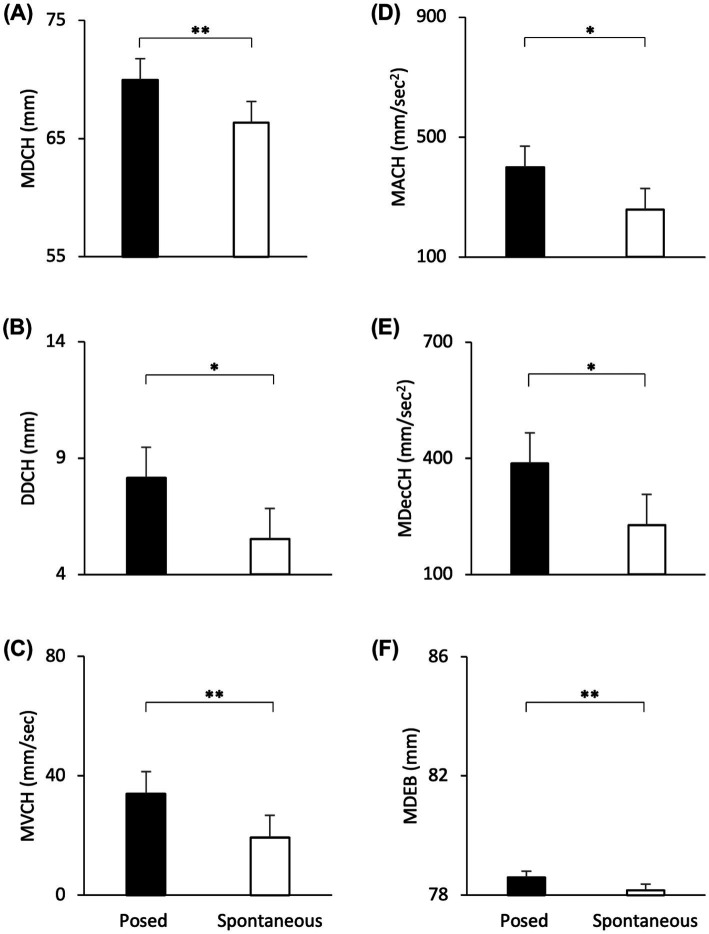
Graphical representation of spatial and speed components of movement in the lower part (i.e., Cheilion markers, CH) and upper part (i.e., Eyebrow markers, EB) of the face during Posed and Spontaneous expressions of happiness. **(A)** Maximum Distance (MDCH), **(B)** Delta Distance (DDCH), **(C)** Maximum Velocity (MVCH); **(D)** Maximum Acceleration (MACH); **(E)** Maximum Deceleration (MDecCH); **(F)** Maximum Distance (MDEB). Error bars represent Standard Error. Asterisks indicate statistically significant comparisons (* = *p* < 0.01; ** = *p* < 0.001).

The Linear Mixed Effect Models revealed a significant effect of Condition with an increase of all spatial and velocity parameters when the participants performed a posed smile, compared to when they smiled spontaneously [MD: *F*_(1,16)_ = 17.721, *p* < 0.001, *VS-MPR* = 75.614, *R*^2^ = 0.843; DD: *F*_(1,16)_ = 9.901, *p* < 0.01, *VS-MPR* = 11.615, *R*^2^ = 0.501; MV: *F*_(1,16)_ = 16.966, *p* < 0.001, *VS-MPR* = 64.217, *R*^2^ = 0.559; MA: *F*_(1,16)_ = 9.283, *p =* 0.009, *VS-MPR* = 8.908, *R*^2^ = 0.481; MDec: *F*_(1,16)_ = 12.146, *p =* 0.004, *VS-MPR* = 17.990, *R*^2^ = 0.521; Figures 4A–E]. None of the temporal parameters revealed significant differences through conditions (all *p*_s_ > 0.05).

##### Upper part of the face—eyebrows

The Linear Mixed Models revealed a significant effect of Condition with an increase of the Maximum Distance of the Eyebrows when the participants performed a posed smile, compared to when they smiled spontaneously [MD: *F*_(1,16)_ = 20.613, *p* < 0.001, *VS-MPR* = 137.386, *R*^2^ = 0.998; Figure 4F]. Velocity and temporal parameters did not result statistically significant (all *p*_s_ > 0.05).

#### Correlations between IRI and kinematics measures

Strong (*r* > 0.7) and positive correlations emerged between IRI measurements and kinematic parameters, but only in the lower part of the face (i.e., Cheilion markers). In particular, two correlations between spatial (MD) and velocity (MV) kinematic parameters were found on the F subscale for the Spontaneous condition (Table 1).

**Table 1:** Pearson’s Correlation between IRI [Cognitive component of empathy, COG: perspective taking (PT) and fantasy (FS) scales; Emotional component of empathy, EMO: empathic concern (EC) and personal distress (PD) scales] and kinematic measures [Maximum Distance (MDCH), Delta Distance (DDCH), Maximum Velocity (MVCH); Maximum Acceleration (MACH); Maximum Deceleration (MDecCH)] of posed and spontaneous expressions.

Condition	IRI		MD CH	DD CH	MV CH	MA CH	MDec CH
Posed	COG	PT	−0.019	−0.524	−0.372	−0.326	−0.297
		FS	0.691	0.041	−0.192	0.246	0.588
	EMO	EC	0.632	0.493	0.227	0.297	0.608
		PD	0.232	0.666	0.418	0.436	0.454
Spontaneous	COG	PT	0.113	−0.155	−0.322	−0.250	−0.186
		FS	**0.772**	0.668	**0.802**	−3.368	−0.347
	EMO	EC	0.467	0.247	0.543	−0.156	−0.141
		PD	0.038	0.062	0.406	0.121	0.137

Strong correlations (*r* > 0.7) are shown in bold.

### Interim discussion

Results from the Linear Mixed Effects Models indicate that posed smiles were performed with larger amplitude, higher peak velocity and deceleration compared to spontaneous expressions. Moreover, we found that posed expressions entailed an increased distance of the Eyebrows. This seems to suggest that activating the Voluntary Pathway on command to perform a posed expression influenced not only the lower part of the face – as expected, but also the upper part of the face. This might be considered surprising in light of the fact that the upper-face is mainly under control of subcortical brain areas. However, studies by Ross and colleagues have highlighted the importance of the upper-face in all expression production (Ross et al., 2007a, 2007b). Correlation analyses with an index of empathic behavior (IRI) showed that the adoption of video clips extracted from comedies activated wider and quicker spontaneous expressions in people who tend to step into the shoes of fictional characters (Fantasy Scale). To further investigate the impact of different induction methods, we run a second experiment.

## Experiment 2

We mirror the emotions of other people even before we become aware of the emotion we are experiencing. The unconscious mimicry of emotions occurs in a similar way to that of motor actions (Frith and Frith, 2023). In this experiment, we evaluated the direct effect of Motor Contagion (i.e., the automatic reproduction of the motor patterns of another individual) on the spontaneous expression of happiness. While in Experiment 1 happiness was induced with movie scenes showing professional actors who performed hilarious scenes without exhibiting smiling faces, in Experiment 2 we selected videos from YouTube in which people were shot frontally while expressing uncontrollable laughter. Posed expressions were tested with the same procedure adopted in Experiment 1.

### Participants

Twenty-five participants (16 females, 9 males) aged between 20 and 29 years (*M*_age_ = 23.01, *SD* = 2.13) voluntarily participated in this study.

### Stimuli

For the Spontaneous condition we adopted *N* = 3 emotion-inducing videos extracted from YouTube (see Validation Study). Videoclips lasted an average of 49 s (video 1 = 31 s; video 2 = 57 s; video 3 = 59 s). While in Experiment 1 videos were longer because the actor needed more time to deliver the hilarious joke, in Experiment 2 the videos were shorter because we isolated only the expressions of happiness. As a result, the time available for participants to spontaneously smile was shorter. We therefore increased the number of stimuli from two to three to collect enough observations. Each video was presented once without repetition to avoid possible habituation effects. Participants rated the intensity of the emotion felt while watching the videos at the end of each presentation to assess that the induction method we adopted was efficient. As for Experiment 1, all the stimuli were judged to have above-average intensity (7) and valence (7) on a 9-point Likert scale.

### Validation study

To select the most appropriate stimuli for the Experiment, we conducted a preliminary online validation study on Qualtrics with 58 healthy volunteers (44 females, 13 males, 1 non-binary; age = 18–60 years). Participants were shown a brief sequence of *N* = 4 videos, and they had to rate how happy they felt after each video clip through a 9-point Likert scale. In addition, they were required to rate valence (positive vs. negative) using a computerized version of the Self-Assessment Manikin—SAM (Bradley and Lang, 1994). Between video clips, participants watched a neutral image chosen by the International Affective Picture System (IAPS; Lang et al., 1997) for 3 s to ensure that emotion induction was not transmitted from one video clip to the next. The happiness scores of each video clip were significantly higher than the midpoint of the scale (i.e., 5; all *p*_s_ < 0.05) and we selected the three video clips with the highest scores on the Likert and the SAM scales for Experiment 2.

### Results

Participants performed at least three expressions of happiness in the Spontaneous condition (range 3–5) and three in the Posed condition.

#### Linear mixed effect models: spontaneous vs. posed lower part of the face—Cheilions

**Figure 5:**
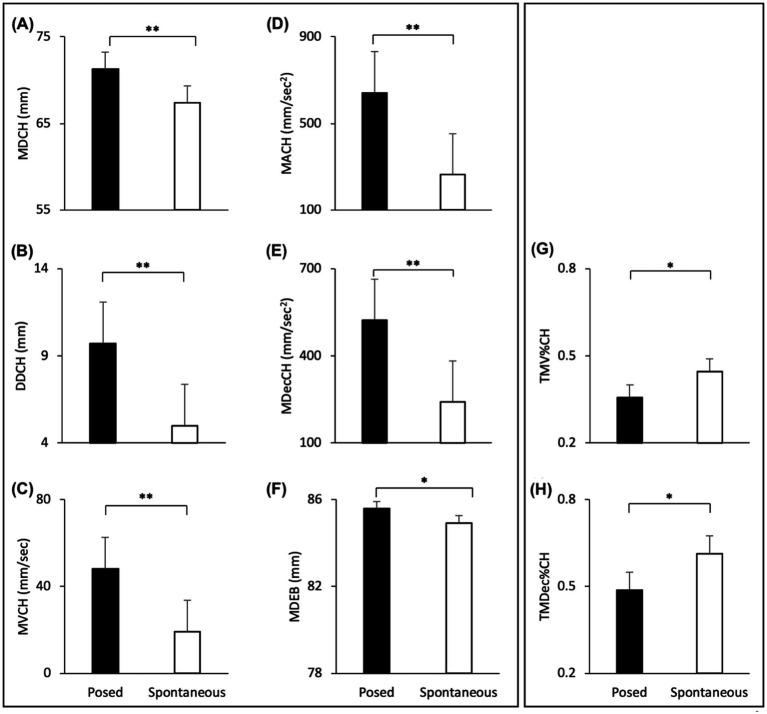
Graphical representation of spatial and speed components of movement in the lower part (i.e., Cheilion markers) and upper part (i.e., Eyebrow markers) of the face during Posed and Spontaneous expressions of happiness. **(A)** Maximum Distance (MDCH), **(B)** Delta Distance (DDCH), **(C)** Maximum Velocity (MVCH); **(D)** Maximum Acceleration (MACH); **(E)** Maximum Deceleration (MDecCH); **(F)** Maximum Distance of the Eyebrows (MDEB); **(G)** Time to Maximum Velocity (TMV%CH); **(H)** Time to Maximum Deceleration (TMDec%CH). The results shown in the left panel **(A-F)** replicate results from Experiment 1 (see Figure 4). The temporal variables shown on the right panel **(G,H)** showed significant differences only in Experiment 2. Error bars represent Standard Error. Asterisks indicate statistically significant comparisons (*p < 0.01; **p < 0.001).

The Linear Mixer Effect Models revealed a significant effect of Condition with an increase of all spatial and velocity parameters when the participants performed a posed smile, compared to when they smiled spontaneously [MD: *F*_(1,24)_ = 55.241, *p* < 0.001, *VS-MPR* = 203200.396, *R*^2^ = 0.889; DD: *F*_(1,24)_ = 72.352, *p* < 0.001, *VS-MPR* = 1,911,000, *R*^2^ = 0.663; MV: *F*_(1,24)_ = 133.321, *p* < 0.001, *VS-MPR* = 547,600,000, *R*^2^ = 0.764; MA: *F*_(1,24)_ = 41.907, *p* < 0.001, *VS-MPR* = 25187.354, *R*^2^ = 0.674; MDec: *F*_(1,24)_ = 36.120, *p* < 0.001, *VS-MPR* = 8779.579, *R*^2^ = 0.643; Figures 5A–E]. In addition, posed smiles reached an earlier velocity peak, followed by an earlier deceleration peak [TMV%: *F*_(1,24)_ = 5.661, *p* = 0.026, *VS-MPR* = 3.916, *R*^2^ = 0.168; TMDec%: *F*_(1,24)_ = 6.747, *p* = 0.016, *VS-MPR* = 5.585, *R*^2^ = 0.117; Figures 5G,H]. None of the remaining temporal parameters revealed statistically significant differences between conditions (all *p*_s_ > 0.05).

##### Upper part of the face—eyebrows

The Linear Mixed Models revealed a significant effect of Condition with an increase of the Maximum Distance of the Eyebrows when participants performed a posed smile, compared to when they smiled spontaneously [MD: *F*_(1,24)_ = 10.278, *p* = 0.004, *VS-MPR* = 17.424, *R*^2^ = 0.986; Figure 5F]. Velocity and temporal parameters did not result statistically significant (all *p*_s_ > 0.05).

#### Correlations between IRI and kinematics measures

No strong correlations emerged between the IRI subscales and kinematic parameters.

### Interim discussion

Results from Experiment 2 indicate that posed expressions of happiness were performed with larger smile amplitude, higher peak velocity, acceleration, and deceleration compared to spontaneous expressions, thus replicating results from Experiment 1. Moreover, posed smiles performed in Experiment 2 differed in temporal terms (i.e., an earlier velocity peak, followed by an earlier deceleration peak) with respect to spontaneous expressions. As regards the Eyebrows, results confirmed those found in Experiment 1 (i.e., increased distance for posed compared to spontaneous expressions). In this experiment (direct method of happiness induction), we expected high correlations with the emotional component of empathy (subscales EC + PD), but analyses showed no strong correlations. This null result might be ascribed to the fact that video clips showed a positive emotion while the subscales measure reactions such as concern and anxiety (see also a criticism recently raised against the two-factor analysis of the IRI; Chrysikou and Thompson, 2016).

## Comparison analysis (Experiment 1 *vs.* 2)

Finally, we run a comparison analysis to test the impact of direct vs. indirect emotional induction methods on the expression of spontaneous and posed happiness.

### Linear mixed analysis: posed vs. spontaneous and Experiment 1 vs. 2 lower part of the face—Cheilions (CH)

Maximum Distance (MD) | A significant main effect of Condition was found [*F*_(1,40)_ = 62.420, *p* < 0.001, *VS-MPR* = 16,520,000, *R*^2^ = 0.869]. Posed expressions were wider than spontaneous smiles (70.630 and 66.874 mm, respectively).

**Figure 6:**
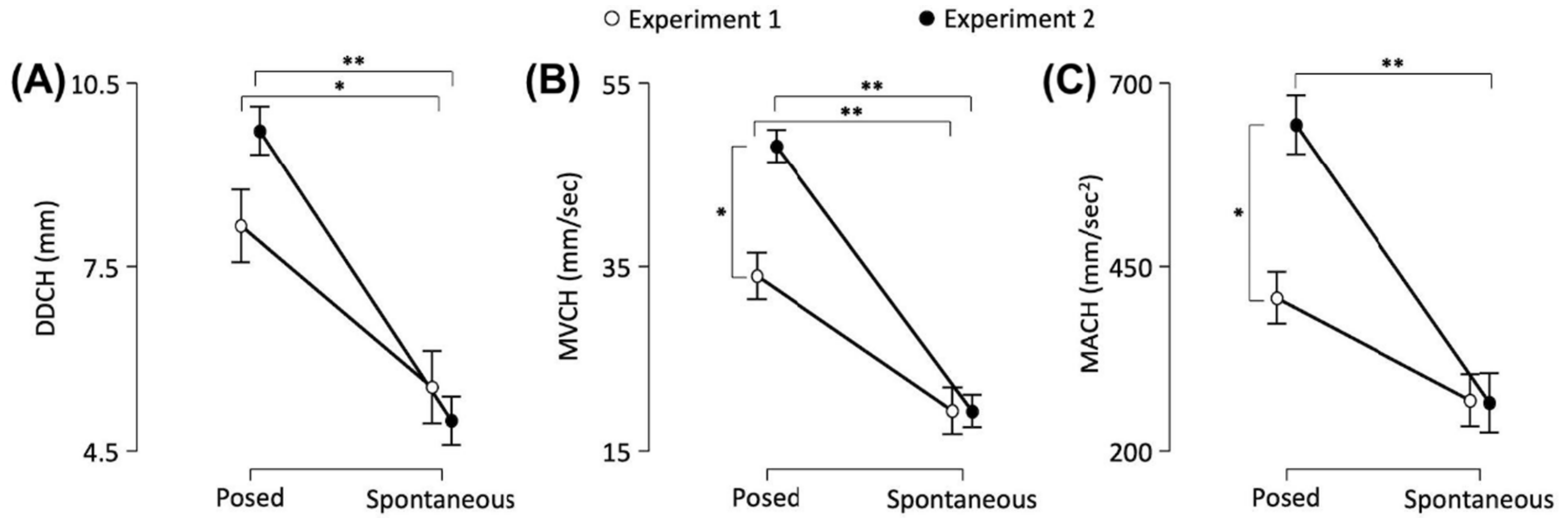
The graphs show: **(A)** the Delta Distance (DD), and peaks of **(B)** Velocity (MV) and **(C)** Acceleration (MA) in the lower part (i.e., Cheilion markers, CH) of the face. Error bars represent Standard Error. Asterisks indicate statistically significant comparisons (*p < 0.01; **p < 0.001).

Delta Distance (DD) | A significant main effect of Condition was found [*F*_(1,40)_ = 58.409, *p* < 0.001, *VS-MPR* = 7,640,000, *R*^2^ = 0.597]. The interaction between Experiment and Condition was also statistically significant [*F*_(1,40)_ = 4.777, *p* = 0.035, *VS-MPR* = 3.151]. *Post hoc* comparisons revealed that posed smiles performed in Experiment 1 had a larger range than spontaneous smiles (8.159 mm vs. 5.532 mm, respectively; *p* = 0.006). The same occurred for Experiment 2: posed smiles had a larger range than spontaneous smiles (9.719 mm vs. 4.988 mm, respectively; *p* < 0.001; Figure 6A).

Maximum Velocity (MV) | A significant main effect of Condition was found [*F*_(1,40)_ = 106.536, *p* < 0.001, *VS-MPR* = 17,150,000,000, *R*^2^ = 0.706]. The interaction between Experiment and Condition was also significant [*F*_(1,40)_ = 11.314, *p* = 0.002, *VS-MPR* = 33.842]. Post hoc comparisons revealed that posed smiles performed in Experiment 1 had a higher peak of Velocity than spontaneous smiles (33.975 mm/s vs. 19.311 mm/s, respectively; *p* < 0.001). The same occurred for Experiment 2: posed smiles had a higher peak of Velocity than spontaneous smiles (48.121 mm/s 19.278 mm/s, respectively; *p* < 0.001). Moreover, posed smiles performed in Experiment 2 showed an increased Maximum Velocity with respect to Experiment 1 (*p* = 0.002; Figure 6B).

Maximum Acceleration (MA) | A significant main effect of Condition was found [*F*_(1,38.2)_ = 37.203, *p* < 0.001, *VS-MPR* = 55661.495, *R*^2^ = 0.544]. The interaction between Experiment and Condition factors was also significant [*F*_(1,38.2)_ = 8.61, *p* = 0.006, *VS-MPR* = 12.606]. *Post hoc* comparisons revealed that posed smiles performed in Experiment 2 showed an increased peak of Acceleration than spontaneous smiles (643.438 mm/s^2^ vs. 265.036 mm/s^2^, respectively; *p* < 0.001). Moreover, the peak of Acceleration was significantly higher for posed expressions in Experiment 2 than in Experiment 1 (643.438 vs. 407.166 mm/s^2^, respectively; *p* = 0.003; Figure 6C).

Maximum Deceleration (MDec) | A significant main effect of Condition was found [*F*_(1,40)_ = 41.066, *p* < 0.001, *VS-MPR* = 183628.817, *R*^2^ = 0.587]. The Maximum Deceleration was higher when the expression was posed than when it was spontaneous (429 and 219 mm/s^2^, respectively). The interaction between Experiment and Condition factors was not significant (*p* > 0.05).

Time to Maximum Acceleration (TMA%) | A significant main effect of Condition was found [*F*_(1,40)_ = 5.309, *p* = 0.024, *VS-MPR* = 4.107, *R*^2^ = 0.069]. The Maximum Acceleration was reached earlier during posed than spontaneous expressions (28.8 and 36.7%, respectively). The interaction between Experiment and Condition factors was not significant (*p* > 0.05).

Time to Maximum Deceleration (TMDec%) | A significant main effect of Condition was found [*F*_(1,40)_ = 7.430, *p* = 0.008, *VS-MPR* = 9.522, *R*^2^ = 0.123]. The Maximum Deceleration was reached earlier during posed than spontaneous expressions (51.3 and 55.4%, respectively). The interaction between Experiment and Condition factors was not significant (*p* > 0.05).

The effects on TMD% and TMV% were not statistically significant (all *p*_s_ > 0.05).

#### Upper part of the face—eyebrows

Maximum Distance (MD) | A significant main effect of Condition was found [*F*_(1,40)_ = 17.299, *p* < 0.001, *VS-MPR* = 257.177, *R*^2^ = 0.993]. Even in the upper part of the face, posed expressions were wider than spontaneous ones (82.083 and 81.543 mm, respectively). None of the remaining parameters was significant (all *p*_s_ > 0.05).

## Discussion

The purpose of the current study was threefold: (i) to measure the performance of spontaneous and posed expressions of happiness across the horizontal axis (i.e., mouth vs. eyebrows); (ii) to test the impact of direct and indirect emotional induction methods on the expression of spontaneous and posed happiness; (iii) to explore the correlation between induction methods and individual differences. To this aim, 3-D motion analysis combined with FACS coding was applied to the study of spontaneous and posed dynamic emotional facial expressions by considering upper and lower parts of the face. Specifically, we analyzed spatial, speed and temporal components of the movement of the Cheilions (i.e., corners of the mouth) and the Eyebrows. Then we adopted two different strategies to induce spontaneous expressions of happiness: observation of hilarious scenes acted by professional actors that induced happiness without showing happy faces (i.e., indirect Emotion Induction, Experiment 1) or (ii) hilarious faces shot frontally (i.e., direct Motor Contagion, Experiment 2). Finally, we correlated the kinematic results with the IRI questionnaire which allows a multi-dimensional assessment of different empathy components. Results from the two Experiments provided stable and reliable 3-D parameters to characterize and distinguish between spontaneous and posed smiles. With regard to the second objective, the results of the comparison analysis (Experiment 1 vs. 2) showed no effect on spontaneous expressions, which appeared fixed regardless of the type of induction adopted (direct or indirect) – as in the case of Dorian Gray’s face, which appears unalterable. Finally, the correlation analysis showed that the cognitive component of empathy seems to play a role when adopting an indirect induction method.

### Posed vs. spontaneous

Consistent results from the two Experiments demonstrated that facial movements provide relevant details to characterize and distinguish between spontaneous and posed expressions. In line with our predictions, results revealed that the speed and amplitude of the mouth as it widens into a smile are greater in posed than spontaneous happiness. Recently, Sowden et al. (2021) demonstrated, using facial landmarks, that speed of facial movements differentiates deliberate expressions of happiness. These findings confirm and extend previous literature (Schmidt et al., 2006, 2009; Guo et al., 2018; Sowden et al., 2021), by showing that performing a fake smile entails a speeded choreography of amplified movements in the lower part of the face. Interestingly, we also found that posed expressions reliably produced an increased distance of the Eyebrows. This seems to suggest that activating the Voluntary Pathway on command to perform a posed expression influenced not only the lower part of the face—under voluntary control of the motor system, but also the upper part of the face. The upper-face muscles (eyes areas) are mainly controlled by subcortical and extrapyramidal systems, but our results might suggest the partial involvement of the cortical motor system as well. This finding, if confirmed, would explain the lack of reliability for the so-called Duchenne marker (i.e., the wrinkling of the skin to produce crow’s feet) and calls for greater caution when studying posed expressions.

### Emotional induction vs. motor contagion

Linear Mixed Analysis on the two Experiments showed a lack of kinematic specificity for spontaneous expressions induced with the emotional induction and motor contagion. This might suggest that both adopted methods were effective in eliciting a genuine emotion mediated by the automatic Involuntary Pathway and therefore free from manipulation effects. Instead, a significant facilitation effect in terms of speed and acceleration was shown for the posed expressions produced in Experiment 2 compared to Experiment 1, despite the fact that the adopted stimulus and task were identical in both experiments. In this second experiment, posed expressions were preceded by observation of smiling faces during the Spontaneous block (i.e., direct induction method). This facilitation effect was likely due to the entrainment of the previously felt emotion (i.e., carry over effect): posed expressions were facilitated when preceded by videoclips inducing motor contagion. Future research is however needed to experimentally manipulate this hypothesis and eventually confirm our interpretation. In general, our data suggest great caution in designing experimental protocols suitable for studying emotion expressions because inducing motor contagion might have affected the mood of participants, therefore altering the production of voluntarily controlled expressions.

Given the central importance of the effectiveness of induction methods in designing experimental work in the field, it is reassuring to note that both methods were equally effective in eliciting comparable spontaneous expressions. Notably, the correct selection of an induction method may be critical when experimental subjects present with cognitive impairments that compromise the effective engagement with the stimuli (Levenson et al., 2008). The present study is among the first to offer some insight into this aspect, by comparing the effectiveness of two different procedures at the kinematic level. These results might contribute to a growing literature on the elicitation of emotion (Coan and Allen, 2007) and the mechanisms that allows the attribution of emotional meaning to others’ facial expressions. According to the models of sensorimotor and embodied simulation (Goldman and Sripada, 2005; Niedenthal, 2007; Gallese and Sinigaglia, 2011; Atee et al., 2017; Sessa et al., 2022), a mechanism of internal simulation is necessary for emotion processing. Social appraisal theories, instead, typically see actions as generated from evaluations (appraisals) of stimulus meaning (Scherer, 2009) and interaction goals (Hess and Fischer, 2013). These two frameworks, often presented as competitive, can in fact be reconciled (Palagi et al., 2020), as it occurred in the broad domain of motor control, where mirroring and mentalizing systems have found common ground (Becchio et al., 2012; Zhang et al., 2024). Recently, indeed, embodiment theories have incorporated the role of context and goals as parameters that can control the extent and form of motor involvement (Zwaan, 2014; Barsalou, 2017; Borghi et al., 2018; Winkielman et al., 2018). Our stimuli probably draw on both mechanisms. While expressions such as the contagious smile can be easily mimicked on the basis of salient contributions from facial features (Experiment 2), the complex stimuli adopted in Experiment 1 that show less explicit expressions (e.g., an ironic scene but no smiling faces) can only gain meaning from contextual cues. In the latter case, emotional expression was probably mediated by elements of emotion understanding, a cognitive level higher than the simple emotional resonance activated by motor contagion. Interestingly, both of our induction methods were effective in activating target expressions, but only motor contagion produced a long-term effect, visible in the subsequent task (carry over effect). This could stem from the fact that the different forms of induction probably recruited different mechanisms with different levels of automaticity and cognitive engagement.

### Interindividual variability

Correlation analyses showed a positive correlation between spatial and speed kinematic parameters of the mouth recorded during the Spontaneous condition and the score on the Fantasy Scale (i.e., tendency to project oneself into the place of fictional characters) only when emotional induction was prompted by professional actors (Experiment 1). The adoption of video clips extracted from comedies activated a wider and speeded smile in people who tend to empathize with fictional characters. As expected, the cognitive component of empathy seems to play a role when adopting an indirect induction method rather than a direct technique.

Overall, these data on induction methods show both a general effect (a fixed spontaneous expression is produced regardless of the stimulus observed) and a specific effect (spontaneous smiling changes on an individual level, depending on whether or not the participant has a predisposition to cognitively empathize with the protagonists of the video). This is an interesting finding for the literature on emotion, which has poorly considered the impact of the different methods used to trigger an emotion and their relation with interindividual variability (for a review see Salas et al., 2012).

The reliance on a fixed order for spontaneous and posed expression – although conceived to hide the true purpose of the study (Perusquía-Hernández et al., 2019; Sowden et al., 2021), might not fully capture the complexity of real-world emotional expression. Future research could explore more ecologically valid settings and procedures to examine how posed and spontaneous expressions differ in everyday social interactions. In addition, individual differences—such as age—may influence expressive behavior and deserve further examination. Despite these limitations, the current findings offer valuable insights with potential translational applications. For instance, understanding the nuanced differences between posed and spontaneous expressions could inform the development of more realistic and emotionally responsive AI systems and virtual avatars. Future research should explore these possibilities, particularly in applied contexts such as mental health assessment, human-computer interaction, and affective computing.

## Conclusion

This research adopted a 3-D optoelectronic system together with a FACS coding to detect and distinguish spontaneous and posed emotion expressions, attesting that a multi-level methodological approach can allow to capture the elusive dimensions of a dynamic smile (for a review, see Straulino et al., 2023a). To summarize, our results show that the spontaneous expression of happiness seems to be an individual signature, a fingerprint derived from subcortical circuits (i.e., Involuntary Pathway) that cannot be altered by different forms of elicitation, and nonetheless show a degree of variability on the basis of personal dispositions. These findings might aid in the development of diagnostic tools for social cognition and prepare the groundwork for future studies in exploring the neural bases of emotional performance.

## Data Availability

The datasets presented in this study can be found in online repositories. The names of the repository/repositories and accession number(s) can be found in the article/Supplementary material.
